# Gallbladder Volvulus Masquerading as Gastric Volvulus: A Diagnostic Challenge in an Elderly Patient With Complex Abdominal Anatomy

**DOI:** 10.7759/cureus.80139

**Published:** 2025-03-06

**Authors:** Paige O Daly, Kaylie Ward, Cole Faulkner, Brian Lenczewski

**Affiliations:** 1 Research, Edward Via College of Osteopathic Medicine, Blacksburg, USA; 2 Trauma Surgery, Riverside Health System, Newport News, USA

**Keywords:** acute abdominal pain, ct imaging, diagnostic laparoscopy, gallbladder volvulus, gangrenous cholecystitis, gastric volvulus, hiatal hernia, misdiagnosis

## Abstract

An 88-year-old female presented to the emergency department with epigastric pain and vomiting, initially diagnosed with gastric volvulus due to a large hiatal hernia. Intraoperative findings revealed gallbladder volvulus instead, leading to gangrenous cholecystitis. This case highlights the diagnostic challenges of gallbladder volvulus, its potential to mimic other acute abdominal conditions, and the importance of considering this rare entity in elderly patients with complex medical histories. The report emphasizes the value of maintaining a high index of suspicion and the role of diagnostic laparoscopy in clarifying complex abdominal pathologies.

## Introduction

Gallbladder volvulus, also known as torsion of the gallbladder, is a rare and potentially life-threatening condition characterized by the rotation of the gallbladder around its mesentery, leading to occlusion of the cystic duct and artery [1]. This condition is exceptionally uncommon, with fewer than 500 cases reported in the literature, predominantly affecting elderly women [2].

The pathophysiology of gallbladder volvulus is closely linked to anatomical variations. Typically, the gallbladder is firmly anchored to the liver, making mobilization highly improbable. However, in cases of gallbladder volvulus, patients often have a "floating gallbladder," which lacks normal peritoneal attachments and possesses an abnormally long, mobile mesentery [3]. This anatomical anomaly significantly increases the risk of torsion.

Another crucial risk factor for gallbladder volvulus is the presence of a hiatal hernia. This condition alters the anatomical positioning and dynamics within the abdominal cavity by modifying intra-abdominal pressure and mechanical forces, potentially displacing the gallbladder from its usual position under the liver [4]. The combination of these factors creates an environment conducive to gallbladder mobility and subsequent volvulus.

The rarity of gallbladder volvulus, combined with its nonspecific presentation, often leads to delayed diagnosis or misdiagnosis, which can lead to necrosis, perforation, peritonitis, and even death. It can present similarly to acute cholecystitis or other acute abdominal emergencies, making preoperative diagnosis challenging. This case report describes an instance of gallbladder volvulus initially misdiagnosed as gastric volvulus in an elderly female with a history of a hiatal hernia. This diagnostic ambiguity underscores the importance of maintaining a broad differential diagnosis in acute abdominal presentations, particularly in elderly patients with complex medical histories.

## Case presentation

An 88-year-old female presented to the emergency department with acute epigastric abdominal pain that began the previous evening after consuming a fruitcake. The patient reported 6-8 episodes of non-bloody, bilious emesis overnight. Her past medical history was significant for hypertension, gastroesophageal reflux disease, hiatal hernia, hyperlipidemia, anxiety, skin cancer, diverticulitis, and a previous gastrointestinal bleed. She denied alcohol, tobacco, or other drug use.

The patient denied fever, chills, shortness of breath, chest pain, constipation, diarrhea, and dysuria. Her vitals included a temperature of 98.2°F, a heart rate of 59 bpm, 14 beats/min respiratory rate, 131/58 mmHg blood pressure, and 96% SpO_2_ on room air. Upon physical examination, the patient was tachycardic with a regular rhythm and normal pulse. Pulmonary effort was normal with normal breath sounds. Mucous membranes were dry. Abdominal examination revealed a flat, soft abdomen with normal bowel sounds and moderate epigastric tenderness.

The CBC results were stable, with a WBC count of 8.0k/mcL, RBC count of 4.04 million/mcL, hemoglobin of 11.6 g/dL, and platelet count of 244k/mcL. Her basic metabolic panel (BMP) revealed that sodium (134 mmol/L), potassium (4.1 mmol/L), chloride (97 mmol/L), anion gap of 9 mmol/L, CO_2_ of 28 mmol/L, blood urea nitrogen (BUN) (20 mg/dL), and creatinine at 0.97 mg/dL were all within normal limits. The hepatic function panel was also normal with a total bilirubin of 0.4 mg/dL, alkaline phosphatase (ALP) of 114 U/L, aspartate aminotransferase (AST) of 17 U/L, alanine transaminase (ALT) of 10 U/L, total protein of 6.5 g/dL, and albumin of 3.7 g/dL. Lipase was 39 U/L. Lactic acid venous was also normal, 0.6 mmol/L. 

**Figure 1:**
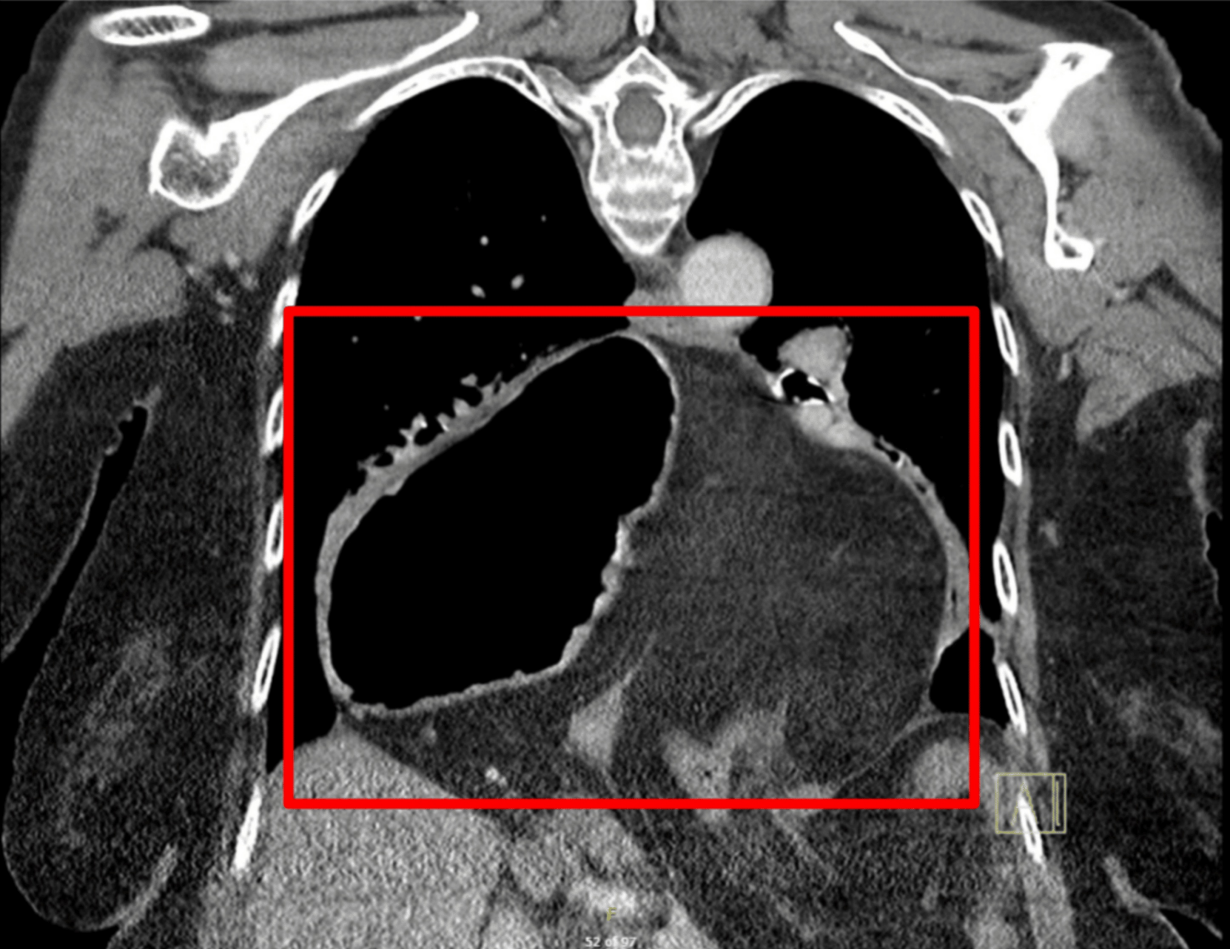
Anterior-posterior chest CT scan with contrast demonstrating a giant hiatal hernia. The CT image reveals a massive hiatal hernia, outlined by the red box, measuring at least 18 cm in transverse diameter. The hernia contents, visible within the thoracic cavity, include the entire stomach, proximal duodenum, and a segment of the transverse colon. This extensive herniation significantly alters the normal anatomical relationships within the chest and upper abdomen, contributing to the complex clinical presentation in this elderly patient.

**Figure 2:**
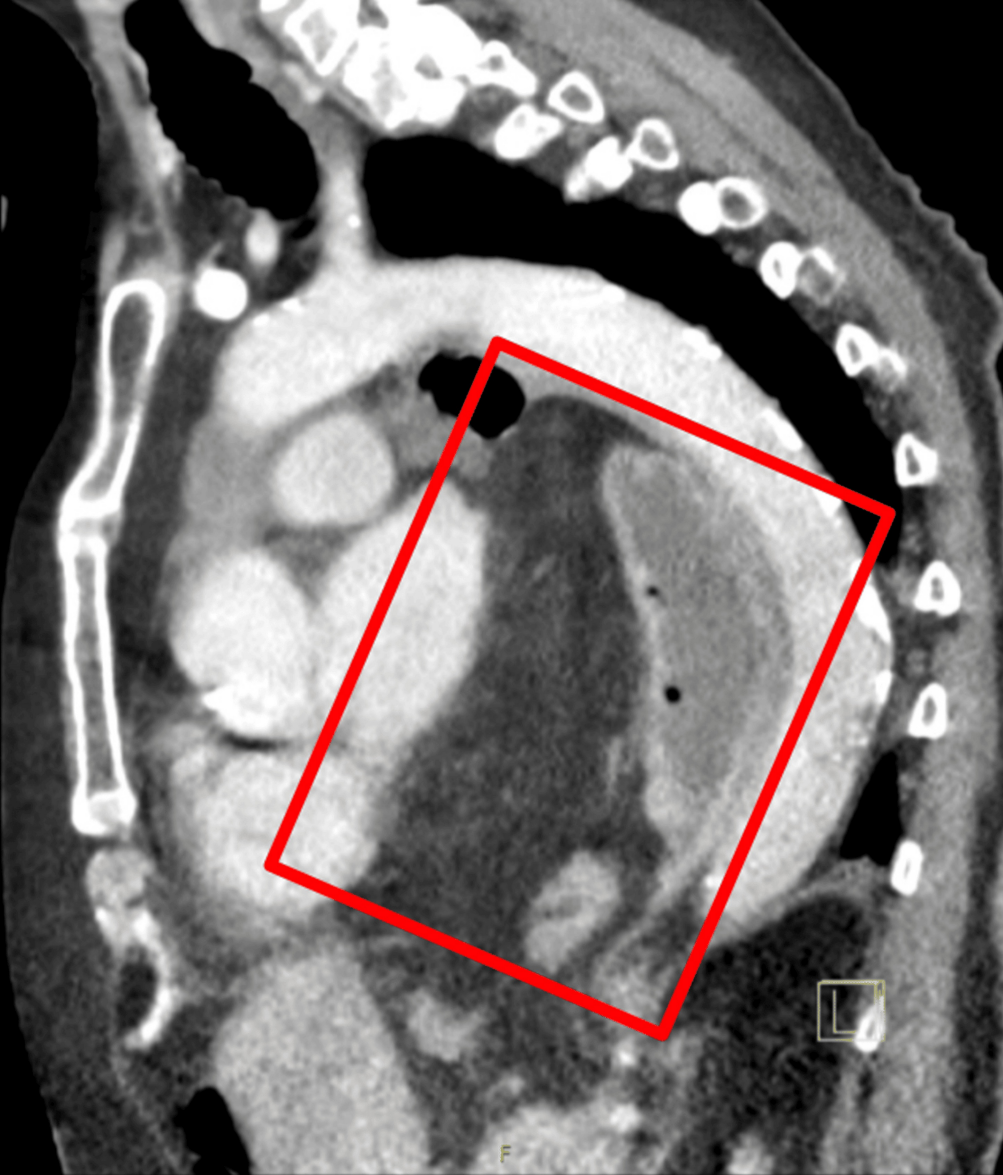
Sagittal chest CT scan with contrast illustrating a giant hiatal hernia. The CT image shows a sagittal view of the chest, with a giant hiatal hernia highlighted by the red box. The hernia is positioned posterior to the heart and anterior to the thoracic/descending aorta. This substantial herniation demonstrates the significant alteration of normal thoracic anatomy, providing insight into the complex presentation and diagnostic challenges in this elderly patient with gallbladder volvulus.

**Figure 3:**
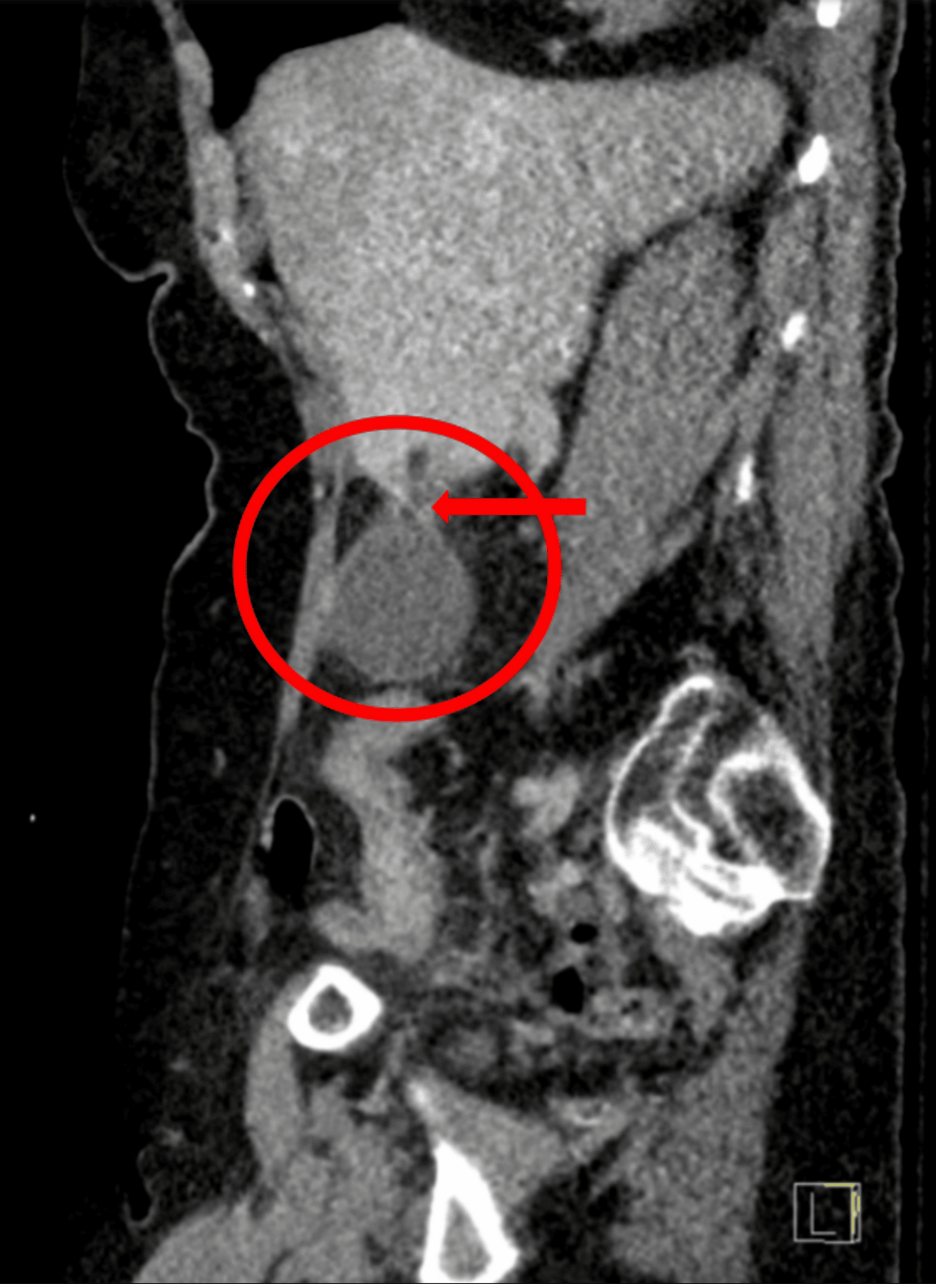
Sagittal abdomen and pelvis CT scan with contrast showing an enlarged gallbladder and potential mesenteric twisting. The CT image displays a sagittal view of the abdomen and pelvis. The red circle highlights an enlarged gallbladder, which appears larger than normal. A red arrow points to an area of potential mesenteric twisting, although it does not exhibit the stereotypical "whirl" sign typically associated with volvulus. This subtle finding, combined with the enlarged gallbladder, illustrates the diagnostic challenge in identifying gallbladder volvulus preoperatively, particularly in the context of complex abdominal anatomy.

**Figure 4:**
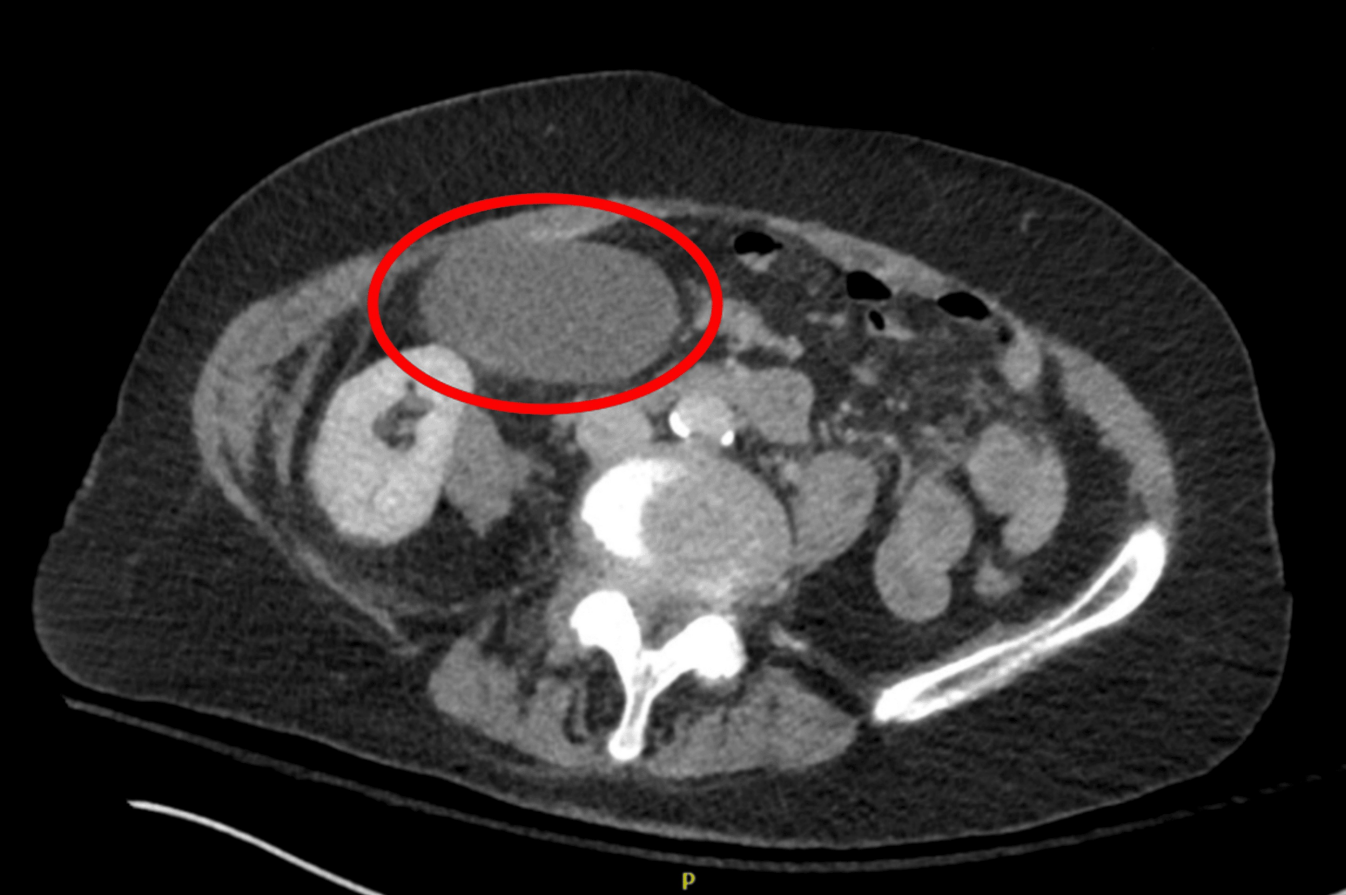
Axial abdomen and pelvis CT scan with contrast revealing an enlarged gallbladder. The CT image shows an axial view of the abdomen and pelvis. A red circle highlights an enlarged gallbladder, which appears significantly larger than normal. This finding, in conjunction with the patient's clinical presentation and other imaging results, illustrates the subtle radiological signs that can be associated with gallbladder volvulus, emphasizing the diagnostic challenge in differentiating this rare condition from other acute abdominal pathologies.

Computed tomography (CT) imaging of the thorax, abdomen, and pelvis with contrast revealed a giant hiatal hernia measuring at least 18 cm in transverse diameter, containing the entire stomach, proximal duodenum, and a segment of the transverse colon (Figures 1, 2). The stomach appeared inverted within the hernia, with the greater curvature superiorly located. This massive hiatal hernia in addition to the appearance of inversion of the stomach led physicians down the path that this was the main cause of the patient's epigastric pain and potentially presenting as an organo-axial volvulus. Abdominal CT showed a distended gallbladder without gallstones, wall thickening, or pericholecystic fluid (Figures 3, 4).

Based on these findings, the initial diagnosis was a type 4 paraesophageal hernia with organo-axial gastric volvulus and suspected gangrenous cholecystitis solely based on CT findings. The patient never met the criteria of Borchardt's triad because the patient had been vomiting, so the diagnosis of a gastric volvulus was made only with the imaging findings. The patient was admitted for diagnostic laparoscopy.

**Figure 5:**
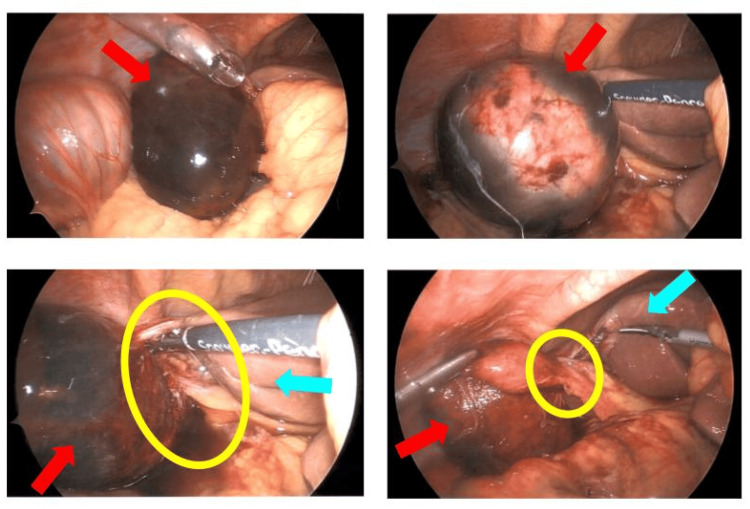
Intraoperative laparoscopic images revealing gallbladder volvulus. This collage of four laparoscopic images demonstrates the key findings during diagnostic laparoscopy. Red arrows indicate the gangrenous gallbladder, which appears dark and discolored due to compromised blood supply. Yellow circles highlight the twisted mesentery, illustrating the volvulus that led to the gallbladder's compromised circulation. Blue arrows point to the liver, providing anatomical orientation and emphasizing the abnormal position of the gallbladder relative to its usual location. These images collectively showcase the unexpected intraoperative discovery of gallbladder volvulus, contrasting with the initial preoperative diagnosis of gastric volvulus.

During the procedure, contrary to the preoperative diagnosis, it was incidentally discovered that the gallbladder had volvulized on itself, resulting in gangrenous cholecystitis (Figure 5). In addition, the stomach was found in the chest, not volvulized, like it was initially thought to be. This proves, once again, that the imaging findings of a gallbladder volvulus are often overlooked for other diagnoses, like the inverted stomach. The only procedure performed on the stomach was repositioning it back into the abdomen. Due to the acute symptoms, the patient was experiencing and a long history of this hiatal hernia, no fundoplication procedure was performed. Fundoplications are mostly considered elective surgeries that the patient must request at a time when they are asymptomatic and not acutely ill.

The incidental finding of the gallbladder being the source of the patient's complaints rather than the stomach also triggered a drastic change in surgical management. The surgeons had to shift quickly into a different type of laparoscopic procedure, in order to carefully remove a necrotic gallbladder with a high risk of perforation and peritonitis. A laparoscopic cholecystectomy was successfully performed to address the gallbladder volvulus, followed by laparoscopic percutaneous endoscopic gastrostomy (PEG) tube placement to ensure that the patient received the necessary nutritional support during her recovery period. The patient recovered well in the following few days and was discharged with a follow-up with her primary care physician. 

## Discussion

Diagnostic challenges and imaging considerations

This case exemplifies the significant diagnostic difficulties associated with gallbladder volvulus. The presence of a large hiatal hernia and the patient's symptoms led to an initial misdiagnosis of gastric volvulus, highlighting the potential for gallbladder volvulus to mimic various acute abdominal conditions. The overlap in clinical presentation between gallbladder volvulus and gastric volvulus, both characterized by acute onset of pain and retching, further complicates the diagnostic process, but they also have distinct clinical features that can aid in differential diagnosis.

Gallbladder volvulus typically presents with acute right upper quadrant (RUQ) abdominal pain, nausea, and vomiting. The pain is often severe and may be associated with signs of acute cholecystitis, such as fever and leukocytosis. Physical examination may reveal RUQ tenderness and a positive Murphy's sign. Gastric volvulus, on the other hand, presents with severe epigastric pain, retching without vomiting, and epigastric distension. The classic triad of Borchardt's triad (severe epigastric pain, retching without vomiting, and inability to pass a nasogastric tube) is highly suggestive of gastric volvulus. 

While CT imaging is valuable in evaluating acute abdominal conditions, it may not always definitively diagnose gallbladder volvulus over other similar presenting conditions. In this case, the distended gallbladder was overshadowed by the more prominent hiatal hernia. The "whirl sign" on CT, indicating a twisted cystic duct, is a key diagnostic feature of gallbladder volvulus but can be subtle and easily overlooked [5]. This sign was not visualized on the patient’s CT. Another key diagnostic feature of previously reported gallbladder volvulus is an edematous and thickened gallbladder wall, which was also not found on this patient’s abdomen and pelvis CT [6]. This case underscores the potential limitations of conventional imaging in complex abdominal pathologies.

To improve preoperative diagnosis, combining CT and ultrasonography (US) can significantly improve the diagnostic accuracy of distinguishing between gallbladder volvulus and gastric volvulus, given their overlapping clinical presentations of acute onset pain and retching. US is often the initial imaging modality for evaluating RUQ pain due to its high sensitivity and specificity for gallbladder pathology. The US can quickly identify gallbladder distension, wall thickening, and the presence of gallstones, which can be indicative of gallbladder volvulus. Performing a CT in addition to the US can prove particularly useful for visualizing the anatomical details and complications associated with the RUQ region. With both being frequently used in the emergency setting, using both imaging techniques might enhance the detection and differentiation of gallbladder volvulus and gastric volvulus, ensuring better patient outcomes.

Other advanced imaging techniques such as magnetic resonance cholangiopancreatography (MRCP) and coronal magnetic resonance imaging (MRI) might provide more detailed visualization in suspected cases too. These modalities could potentially reveal the characteristic signs of gallbladder torsion, such as a distorted gallbladder neck and cystic duct, which may be less apparent on CT scans. The concern for using additional imaging, like MRCP and MRI, preoperatively is the increased risk of the volvulized organ perforated from being necrotic and causing peritonitis due to them not being "quick" imaging. Finally, having the risk of a volvulized organ might make the procedure of an endoscopic retrograde cholangiopancreatography (ERCP) and MRCP difficult to complete due to the organ twisting on itself.

Surgical management and elderly patient considerations

Prompt surgical intervention is crucial in managing gallbladder volvulus to prevent complications such as gangrene and perforation. Laparoscopic cholecystectomy serves as a diagnostic and therapeutic approach. In this case, the intraoperative findings were key to establishing the correct diagnosis and guiding appropriate treatment. The decision to perform a laparoscopic PEG placement demonstrates the importance of addressing potential postoperative nutritional needs in complex cases, particularly in elderly patients, because otherwise PEG placement is not routinely recommended after a standard laparoscopic cholecystectomy.

This case underscores the importance of maintaining a high index of suspicion for atypical presentations of acute abdominal conditions in elderly patients. Comorbidities and anatomical variations can significantly complicate the clinical picture, necessitating a broad differential diagnosis. The presence of a large hiatal hernia in this patient added complexity to the presentation and initial interpretation of imaging studies, emphasizing the need for a comprehensive approach to diagnosis in elderly patients.

In addition, elderly patients, particularly those over 80 years, have a significantly higher rate of postoperative complications, including cardiovascular, pulmonary, and infectious complications. Studies have shown that the complication rate in 90-99-year-old patients undergoing cholecystectomy is notably higher, with increased rates of conversion to open surgery, prolonged hospital stays, and higher mortality rates. Tailored perioperative care, including careful preoperative optimization, vigilant intraoperative monitoring, and intensive postoperative care, is crucial, and given the higher risks, it is essential to have thorough discussions with patients and their families about the potential risks and benefits of surgery. This includes discussing the likelihood of complications, the potential need for intensive care, and the expected recovery trajectory. These factors necessitate a comprehensive and individualized approach to improve outcomes and ensure informed decision-making.

Epidemiology and risk factors

Gallbladder volvulus predominantly affects elderly women, with a peak incidence in the seventh and eighth decades of life [2]. The patient in this case fits the typical demographic profile. The reason behind this condition occurring in this demographic is due to hormonal influences and impaired gallbladder motility. Estrogen has been implicated in the pathogenesis of many gallbladder diseases by increasing the saturation of cholesterol in bile, leading to the formation of gallstones and therefore chronic inflammation. Aging also affects gallbladder motility, leading to impaired emptying and stasis of bile. This also can result in chronic inflammation and fibrosis, further predisposing the gallbladder to torsion. A study by Armistead et al. described a case where a hiatal hernia was also present in a patient with gallbladder volvulus, suggesting a possible association between the two conditions. 

Another major risk factor for gallbladder volvulus includes anatomical variations such as a redundant mesentery, which allows for increased mobility of the gallbladder and the gallbladder adopting the nickname "floating gallbladder". Finally, the presence of a large hiatal hernia in this patient may have also contributed to altered intra-abdominal dynamics, potentially predisposing to gallbladder volvulus. A study by Armistead et al. described a case where a hiatal hernia was also present in a patient with gallbladder volvulus, suggesting a possible association between the two conditions [4].

The rarity of this condition, combined with its potential for severe complications, highlights the need for increased awareness among clinicians. Understanding the risk factors and maintaining a high index of suspicion in elderly patients presenting with acute abdominal pain can lead to more timely diagnoses and improved outcomes.

## Conclusions

This case report underscores the diagnostic challenges associated with gallbladder volvulus, particularly in elderly patients with complex medical histories. The initial misdiagnosis of gastric volvulus highlights the potential for gallbladder volvulus to mimic other acute abdominal conditions, especially in the presence of concurrent anatomical abnormalities such as large hiatal hernias. The case emphasizes the value of diagnostic laparoscopy as a minimally invasive procedure that allows direct visualization of the abdominal cavity, providing a definitive diagnosis when imaging studies are inconclusive. This is particularly valuable in elderly patients, where conditions like gallbladder volvulus may present atypically and imaging may not always be definitive. The case brings attention to the importance of keeping a high index of suspicion for rare conditions like gallbladder volvulus. Recognizing the limitations of imaging studies, such as CT and ultrasound, in these complex cases is essential. While imaging can provide valuable information, it may not always capture the dynamic nature of the volvulus or differentiate it from other conditions like gastric volvulus. The case serves as a reminder of the need for a multidisciplinary approach, involving surgeons, gastroenterologists, and radiologists, to optimize patient outcomes. By sharing this case, we aim to increase awareness among healthcare professionals, potentially leading to more timely diagnoses and improved patient outcomes in similar cases of gallbladder volvulus.
